# Crosstalk between circRNAs and the classic signaling pathways in IBD

**DOI:** 10.1080/15476286.2026.2684781

**Published:** 2026-06-15

**Authors:** Mengmeng Jie, Qiushi Liao, Cheng Liu

**Affiliations:** Department of Gastroenterology, Xinqiao Hospital, Army Medical University, Chongqing, China

**Keywords:** Inflammatory bowel disease, Circular RNA, pathway, intestinal epithelial homoeostasis, biomarker

## Abstract

Inflammatory bowel disease (IBD), which includes Crohn’s disease (CD) and ulcerative colitis (UC), is a chronic inflammatory disorder of the gastrointestinal tract with a complex aetiology involving genetic, environmental (such as diet/lifestyle), and microbiota factors. The exact pathogenesis of IBD remains unclear, and current therapies show limited effectiveness. Circular RNAs (circRNAs) are a novel class of noncoding RNAs that have been implicated in the regulation of various biological processes. Many circRNAs show specific expression profiles in IBD patients and play important roles in IBD pathogenesis through different signalling pathways, such as the Janus kinase/signal transducer and activator of transcription (JAK/STAT) and nuclear factor-κB (NF-κB) pathways. Although research on circRNAs in IBD is still in its early stages, many circRNAs have emerged as potential diagnostic, prognostic biomarkers, and therapeutic targets for IBD. Here, we summarize the molecular functions and underlying mechanisms of circRNAs in IBD and discuss current challenges and future perspectives for clinical applications.

## Introduction

Inflammatory bowel disease (IBD) is a group of chronic inflammatory diseases of the gastrointestinal tract that is associated with poor quality of life and economic burden of patients worldwide. Ulcerative colitis (UC) and Crohn’s disease (CD) are the main types of IBD [1]. The global burden of IBD is projected to increase over the next two decades, driven by rising incidence and prevalence [2]. It is hypothesized that the rapid increase in the number of IBD patients is associated with the Western dietary pattern and modern lifestyle [3]. The exact aetiology of IBD remains complex and multifactorial, involving interactions between genetics, environmental exposures, and the gut microbiota [4;5]. The main therapeutic goal for IBD patients is to induce and maintain disease remission. With the approval of the first biological drug, infliximab, deep remission has been pursued, including endoscopic mucosal healing, histological, and transmural healing [6–8]. The pharmacotherapy of IBD includes traditional drugs (5-aminosalicylates, corticosteroids, and immunomodulators, etc.), biologics (anti-TNF: Infliximab, Adalimumab; anti-α4β7 integrin: Vedolizumab; anti-IL-12/IL-23: Ustekinumab; anti-IL-23: Risankizumab), and small molecules (JAK inhibitors: Tofacitinib, Filgotinib, Upadacitinib; S1P: Ozanimod, Etrasimod) [9–13]. The selection of IBD treatment should be individually determined by various factors, including disease severity, location, complications, patients’ choice, and drug-related factors (medical history, therapeutic drug monitoring, cost, safety, administration, etc.). However, only 40–60% of IBD patients achieve clinical remission after individual treatments [14,15]. Therefore, many studies have reported some reasonable alternatives for refractory and severe IBD patients, including dose optimization, combination therapy with a thiopurine or methotrexate and anti-TNF, and the combination of two biological therapies, such as anti-TNF plus vedolizumab and vedolizumab plus ustekinumab [16,17]. The improvements and breakthroughs in IBD treatment still require more exploration.

Circular RNAs (circRNAs) are a novel class of noncoding RNAs that have attracted significant attention in recent years [18,19]. Unlike their linear counterparts, circRNAs have no 5’ caps or 3’ polarity and are characterized by the covalently closed-loop structure, which results from the back-splicing of pre-mRNAs. Due to the unique structure, circRNAs are more resistant to degradation and more stable than linear RNAs. In addition, circRNAs are highly conserved across different species and display cell-type and tissue-specific patterns [20,21]. At first, circRNAs are considered to be byproducts of splicing errors [22]. With the development of sequencing technology and bioinformatics, an increasing number of circRNAs are validated and have abundant expression [23,24]. Recent studies have demonstrated that circRNAs participate in various diseases, such as cancer, infection, cardiovascular disease, metabolic disease, and autoimmune disease [25–30]. CircRNAs can act as competing endogenous RNAs (ceRNAs) to sponge miRNAs and regulate target gene expression [31,32]. The most classic circRNA, CDR1as/ciRS-7, contains 74 miR-7 seed matches and functions as a miR-7 sponge to regulate the expression of miR-7 targets. Furthermore, circRNAs can interact with RNA-binding proteins (RBPs) at the post-transcriptional level to alter the location or modifications of proteins [33–35]. It is reported that upregulation of circ0006646 is associated with poor prognosis of hepatocellular carcinoma (HCC) and promotes the metastasis of HCC in vitro and in vivo. Mechanistically, circ0006646 directly interacts with nucleolin and stabilizes nucleolin expression by hindering the binding of nucleolin and the E3 ligase tripartite motif-containing 21 (TRIM21). Moreover, the therapeutic value of circ0006646 is confirmed through patient-derived tumour xenograft models [36]. Due to the specific expression pattern and high stability, circRNAs are considered as diagnostic biomarkers, therapeutic targets, prognostic markers, and even vaccines for diseases [37–39].

**Figure 1.**
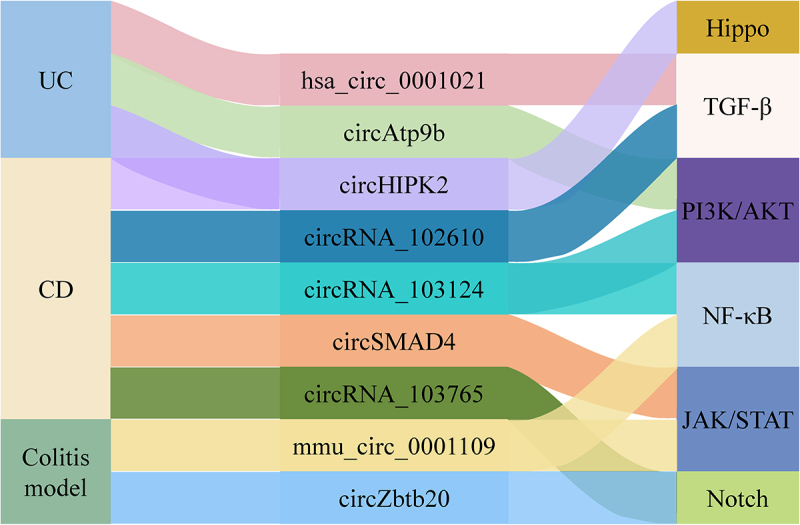
CircRNAs in IBD-related pathways.

Recent studies have demonstrated the association between circRNAs and IBD [40,41]. Qiao YQ et al. have reported the differential expression of circRNAs in the colon tissue of CD patients compared with healthy controls through circRNA microarrays [42]. In total, 163 upregulated circRNAs and 55 downregulated circRNAs were identified in CD patients. It is predicted that has_circRNA_102685 might regulate the pathways associated with IBD, including apoptosis, TLR, and p53 signalling pathways via sponging miR-146. In another study, 155 upregulated and 229 downregulated circRNAs were detected in peripheral blood mononuclear cells (PBMCs) from CD patients compared with healthy controls [43]. Further receiver operating characteristic (ROC) analysis reveals that the area under the curve (AUC) of has_circRNA_004662 is 0.85, indicating that it can serve as a potential biomarker for CD diagnosis. While the expression of has_circRNA_004662 in CD is higher than that in UC (*p* < 0.001), there is no difference between UC and healthy controls (*p* = 1.00). So, circRNA_004662 might be a potential biomarker for distinguishing CD from UC. Yuan et al. reported the differentially expressed circRNAs in UC from the Gene Expression Omnibus dataset (GEO) [44]. A new, predictive nomogram constructed using hsa_circ_0085323 and hsa_circ_0036906 has been proven effective for UC diagnosis [44]. These studies preliminarily confirm that circRNAs participate in the regulation of various IBD-related signalling pathways and may contribute to IBD pathogenesis. In this review, the IBD-related signalling pathways, including the Janus kinase/signal transducer and activator of transcription (JAK/STAT), Wnt, phosphatidylinositol 3‑kinase/AKT (PI3K/AKT), nuclear factor-κB (NF-κB), and Hippo pathways, are discussed, as well as their roles in the pathogenesis of IBD. We also summarize the current research on the role and molecular functions of circRNAs in IBD-related pathways and discuss the potential application in clinical practice (Table 1 and Figure 1). This review will give a new insight into IBD diagnosis and treatment targeting circRNAs and the related pathways.

**Table 1.** CircRNAs involved in IBD.

circRNA	circBase ID	Gene symbol	Disease	Source	Change	Pathway	Function and mechanism	Application	Reference
circRNA_102610	hsa_circ_0000972	MBOAT2	CD	PBMCs	Upregulate	TGF-β	Promotes proliferation and epithelial mesenchymal transition of IECs via sponging hsa-miR-130a-3p	Diagnostic marker, therapeutic target	[43;45]
circRNA_103765	hsa_circ_0071375	KLHL2	CD	PBMCs	Upregulate	Notch	Promotes IECs proliferation and apoptosis via miR-30/DLL4 axis	Diagnostic marker, therapeutic target	[46]
circSMAD4	hsa_circ_0006952	SMAD4	CD	Colon tissues	Upregulate	JAK/STAT	Impairs epithelial barrier integrity via miR-135a-5p/JAK2 axis	Therapeutic target	[47]
circRNA_102685	hsa_circ_0003578	CRIM1	CD	Colon tissues	Upregulate	Toll-like receptor, p53	Sponges miR-146	Diagnostic marker, therapeutic target	[42]
circPRKAR1B	hsa_circ_0008039	PRKAR1B	CD	Colon tissues	Upregulate		Promotes NLRP3 inflammasome-mediated pyroptosis and inhibits autophagy to aggravate colitis via interacting with SPTBN1	Therapeutic target	[48]
hsa_circ_0001666	hsa_circ_0001666	FAM120B	CD	Colon tissues	Upregulate		Unclear	Diagnostic marker	[49]
hsa_circ_0062142	hsa_circ_0062142	TPTEP1	CD	Colon tissues	Upregulate		Unclear	Diagnostic marker	[49]
circRNA_092520	hsa_circ_0001240	NFAM1	CD	PBMCs	Upregulate		Unclear	Diagnostic marker	[43]
circGMCL1	hsa_circ_0055097	GMCL1	CD	Colon tissues	Downregulate		Promotes autophagy and alleviates pyroptosis via miR-124-3p/Annexin 7	Therapeutic target	[40]
circRNA_103124	hsa_circ_0001187	DOPEY2	CD, UC	PBMCs	Upregulate	PI3K/AKT, NF-κB	Promotes cell proliferation and macrophage M1 polarization and inhibits autophagy via hsa-miR-650/AKT2 pathway, and aggravates TNF-α-induced cell apoptosis, inflammation and oxidative stress through the miR-1236-3p/MYD88 axis	Diagnostic marker, therapeutic target	[50–52]
circRNA_103516	hsa_circ_0003692	FNDC3B	CD, UC	PBMCs	Upregulate		Sponges hsa-miR-19b-1-5p	Diagnostic marker	[53]
circSOD2	hsa_circ_0004662	SOD2	CD, UC	Colon tissues, PBMCs	Upregulate		Destroys the intestinal mucosal barrier via miR-378 g/Snail1 axis	Diagnostic marker, therapeutic target	[43,54]
Cdr1as	hsa_circ_0001946	CDR1	CD, UC	Colon tissues	Upregulate		Suppresses renewal and defence of the intestinal epithelium via miR-195	Therapeutic target	[55]
circHIPK2	hsa_circ_0001756	HIPK2	CD, UC	Colon tissues	Upregulate	Hippo	CircHIPK2 enhances the translational efficiency of TAZ and increases the transcription of target genes, namely cellular communication network factor 1 (CCN1) and CCN2, leading to intestinal epithelial growth in colitis	Therapeutic target	[56]
circHIPK3	hsa_circ_0000284	HIPK3	CD, UC	Colon tissues	Downregulate		Promotes intestinal epithelial repair and renewal through sponging miR-29b	Therapeutic target	[57]
circRNA CCND1	hsa_circ_0023303	CCND1	UC	Colon tissues	Downregulate		Suppresses cell apoptosis and inflammatory responses via miR-142-5p/NCOA3 pathway	Therapeutic target	[41]
circHECTD1	hsa_circ_0101590	HECTD1	UC	Colon tissues	Downregulate	AMPK/mTOR	Promotes autophagy to mitigate DSS-induced colitis via miR-182-5p/HuR axis	Therapeutic target	[58]
hsa_circ_0001021	hsa_circ_0001021	AFTPH	UC	Colon tissues	Downregulate	TGF-β	Protects intestinal epithelial barrier via miR-224-5p/smad4	Therapeutic target	[59]
hsa_circ_0007919	hsa_circ_0007919	ABR	UC	Colon tissues	Downregulate		Regulates intestinal epithelial barrier via hsa-let-7a/EPC1 and hsa-miR-138/VIPR1 axis	Diagnostic marker, therapeutic target	[60]
circCDKN2B-AS1	hsa_circ_0008574	CDKN2B-AS1	UC	Colon tissues	Downregulate		Inhibits cell proliferation and epithelial barrier through upregulating Claudin-2	Therapeutic target	[61]
circ_0085323	hsa_circ_0085323	EIF3E	UC	Colon tissues	Upregulate		Aggravates TNFα-induced inflammation and cell apoptosis via miR-495-3p/TRAF3	Diagnostic marker, therapeutic target	[62]
circPABPN1	hsa_circ_0031288	PABPN1	UC	Colon tissues	Upregulate		Suppresses cell autophagy through blocking the bind of HuR to Atg16l1 mRNA and reducing ATG16L1 protein levels	Unclear	[63]
mmu_circ_0001109	mmu_circ_0001109	RTEL1	Colitis model	Colon tissues	Upregulate	JAK/STAT, NF-κB	upregulates the p-STAT3 and p-P65 levels, which further aggravate colitis through JAK/STAT and NF-κB pathways	Unclear	[64]
circAtp9b	–	ATP9B	UC	Plasma	Upregulate	PI3K/AKT/PTEN	Promotes LPS-induced apoptosis via PTEN	Diagnostic marker, therapeutic target	[65]
circZbtb20	mmu_circ_0006298	ZBTB20	Colitis model	ILC3	Upregulate	Notch	Maintains ILC3 homoeostasis via the m6A demethylation of Nr4a1 mRNA	Unclear	[66]
circKcnt2	mmu_circ_0000084	KCNT2	Colitis model	ILC3	Upregulate		Induces ILC3 inactivation and colitis resolution through suppressing IL-17 expression	Unclear	[67]

## JAK/STAT pathway

The JAK/STAT pathway is essential in regulating immune processes, intestinal epithelial homoeostasis, inflammation, cell growth, and differentiation [68–70]. Genetic mutations and dysregulation of the JAK/STAT pathway are associated with various diseases, such as cancer, immune disorders (e.g. rheumatoid arthritis, atopic dermatitis and IBD), and virus infections including COVID-19 [71,72]. JAK proteins are non-transmembrane tyrosine kinases, including JAK1, JAK2, JAK3, and tyrosine kinase (TYK2), of which JAK3 exhibits a predominantly restricted expression pattern in haematopoietic cells. There are seven members of STAT proteins, including STAT1, STAT2, STAT3, STAT4, STAT5A, STAT5B, and STAT6. When cytokines specifically bind to receptors, the receptors get close to each other and form dimers. Then the JAK proteins are activated, and the activating JAKs phosphorylate the intracellular domain of the receptors, which form docking sites for the recruitment of STATs. Subsequently, STATs are phosphorylated by JAKs, resulting in the homodimerization or heterodimerization of phosphorylated STATs. The STAT dimers dissociate from receptors, translocate to the nucleus, and bind to the DNA regulatory elements to regulate the transcription of target genes. For given cytokines, the corresponding JAKs and STATs are specifically activated and regulate different biological processes [73]. In human memory CD4+ T cells, IL-23 induces STAT1, STAT3 and STAT4 phosphorylation, whereas IL-12 predominantly activates STAT4 alone [74]. In mouse Th17 cells, IL-23 signalling via STAT3 and STAT4 promotes Th17 cell survival, expansion, and pathogenic conversion to Th1-like cells [75]. Both IL-12 and IL-23 cytokines also act on human and mouse innate lymphoid cells (ILCs), NK cells, and intestinal epithelial cells (IECs) to regulate antibacterial responses and cytokine secretion [76]. IL-6 activates STAT3 via JAK1, JAK2, and TYK2, promoting cell survival, proliferation, and wound healing in IECs and regulating apoptosis, differentiation, and cancer progression in cancer cells, across human and mouse models [77,78]. In response to Interferon-α (IFN-α) and IFN-β, STAT1 and STAT2 are activated via JAK1 and TYK2. These pathways have been confirmed in human and mouse immune cells, including dendritic cells, macrophages, and T cells, as well as non-immune cells such as fibroblasts and epithelial cells, where they mediate antiviral responses and immune regulation [79]. IFN-γ activates STAT1 and STAT3 through JAK1 and JAK2. This pathway, well characterized in human and mouse macrophages, T cells, and NK cells, participates in antitumor and immune regulation [79]. It has been well studied that cytokines play crucial roles in the immune dysfunction, intestinal inflammation, clinical manifestations, and complications of IBD patients [80]. Cytokines associated with IBD, such as IL-12, IL-23, IL-6, and IFN-γ, exert their effects through JAK/STAT pathway [81]. Anti-IL-12/IL-23 (Ustekinumab) and anti-IL-23 (Risankizumab) antibodies, which target upstream cytokines that activate the JAK-STAT pathway, have been successively approved for the treatment of IBD. Unlike biologics targeting individual cytokines, the small-molecule JAK inhibitors block multiple cytokines and show efficacy in the induction and maintenance therapy for UC and CD. Tofacitinib, preferentially targeting JAK1 and JAK3, was the first approved JAK inhibitor for the moderately to severely active UC, but had no significant efficacy in CD [82,83]. Upadacitinib, the JAK1 selective inhibitor, was approved for the treatment of both UC and CD [84,85].

Many studies have validated that circRNAs may participate in IBD via the JAK/STAT signalling pathway. The differential expression profiles of mouse circRNAs were identified by RNA-seq in dextran sulphate sodium (DSS)-induced colitis and colitis-associated cancer (CAC), of which mmu_circ_0001109 is highly expressed in DSS-induced colitis tissues compared to the normal tissues [64]. The expression of mmu_circ_0001109 was positively associated with p-STAT3 and p-P65 expression. Moreover, mmu_circ_0001109 overexpression upregulated the p-STAT3 and p-P65 levels, which further validated that mmu_circ_0001109 aggravated colitis through JAK/STAT and NF-κB signalling pathways. However, the human homologue of mouse mmu_circ_0001109 showed no similar expression in the tissues mentioned above, possibly owing to low sequence similarities. Zhao et al. demonstrated that circSMAD4 was upregulated in CD patients’ tissues compared with controls and was positively correlated with inflammatory indexes, such as C-reactive protein (CRP) and faecal calprotectin [47]. Meanwhile, poly lactic-co-glycolic acid microspheres (PLGA MSs) carrying si-circSMAD4 were constructed and gavage administered to IL-10 KO mice. The inflammatory infiltration and the levels of pro-inflammatory cytokines including TNF-α, IFN-γ, and IL-17, were reduced after PLGA MSs carrying si-circSMAD4 treatment. The si-circSMAD4 treatment promoted the differentiation of CD4+T cells into Treg cells instead of Th1 and Th17 cells. Furthermore, circSMAD4 upregulated JAK2 expression and subsequent p-STAT3 level by sponging miR-135a-5p to impair intestinal barrier function and promote colitis progression, which was consistent with the function mediated by JAK2/STAT3 signalling pathway. Overall, circSMAD4 impaired intestinal barrier integrity and promoted colitis progression via the miR-135a-5p/JAK2 axis in experimental colitis. PLGA MSs carrying si-circSMAD4 treatment alleviated intestinal inflammation in the colitis mice model. Notably, this study utilized siRNA-mediated knockdown rather than genetic knockout of circSMAD4. To date, no circSMAD4 knockout mouse models have been reported to definitively establish its physiological roles. The development of circSMAD4-deficient mice, such as using CRISPR/Cas9 to delete circularization-specific exons, would provide conclusive validation of its functional necessity and cell-type-specific mechanisms. Such genetic models represent a critical future direction to validate circSMAD4 as a therapeutic target. CircSMAD4 might be a promising therapeutic target for IBD, but further research is needed to explore its clinical application. While therapies targeting circSMAD4 show promise for IBD, numerous questions remain to be addressed, including the roles of circSMAD4 in other cell types, optimal dosage and delivery strategies, long-term safety of genetic or pharmacological circSMAD4 inhibition, and the efficacy and safety of circSMAD4 in human clinical trials.

The JAK/STAT pathway serves as a critical nexus linking circRNA-mediated regulatory networks to the pathogenesis of IBD. CircRNAs such as mmu_circ_0001109 and circSMAD4 modulate intestinal inflammation and epithelial barrier integrity through diverse mechanisms, including miRNA sponging and interaction with RBPs, ultimately influencing immune cell differentiation, cytokine production, and disease progression. These findings highlight the therapeutic potential of targeting circRNA-JAK/STAT axes, as evidenced by siRNA-based interventions in preclinical models. However, clinical translation requires further investigation into species-specific circRNA functions, cell-type-specific roles, optimal delivery systems, and safety profiles in human trials.

## Wnt pathway

The Wnt pathway is a key signalling pathway in developmental processes, such as cell proliferation and differentiation, and it is highly conserved in evolution [86]. Wnt pathway includes canonical pathway (Wnt/β-catenin) and non-canonical pathways (Wnt/planar cell polarity (PCP) and Wnt/Ca^2+^) [87,88]. In canonical Wnt/β-catenin pathway, Wnt proteins, including Wnt1, Wnt2b, Wnt3, Wnt3a and Wnt8a, bind to Frizzled (FZD) and lipoprotein receptor-related protein (LRP) receptors, leading to the prevention of β-catenin degradation mediated by the destruction complex (comprising the scaffold protein Axin, adenomatous polyposis coli (APC), glycogen synthase kinase (GSK3), and casein kinase 1 (CK1)) [89]. Subsequently, β-catenin is accumulated and translocated to the nucleus, then binds to T cell factor (TCF) to initiate the transcription of target genes. When Wnt signalling is off, β-catenin is phosphorylated and degraded by the destruction complex. In non-canonical Wnt signalling, Wnt4, Wnt5a, Wnt5b, Wnt6, Wnt7a and Wnt11 bind with FZD to regulate cell polarity, migration, cytoskeleton organization, and intracellular calcium release [90].

The Wnt/β-catenin signalling pathway is a highly conserved regulator of tissue homoeostasis and stem cell maintenance across multiple organ systems. Beyond its well-characterized role in embryonic development, canonical Wnt signalling governs the self-renewal of various adult stem cell populations, including intestinal stem cells (ISCs), mesenchymal stem cells (MSCs), and embryonic stem cells [91,[89]]. Aberrant activation of this pathway disrupts tissue homoeostasis, leading to unchecked proliferation and the accumulation of stem-like cells that can serve as precursors to malignant transformation [92]. Emerging evidence indicates that Wnt signalling intimately links cell identity with immunological function. In the immune system, Wnt/β-catenin signalling regulates T cell development, differentiation, and memory formation. Recent studies have revealed that WNT signalling components, particularly Wnt5a and β-catenin, play pivotal roles in antigen processing, presentation, and subsequent T cell activation [93–95].

Studies have demonstrated that Wnt signalling is involved in a wide range of biological processes and regulates multiple cellular functions, including those of Paneth cells, MSCs, ISCs, and immune cells in patients with IBD [96–98]. In general, inactivation or suppression of Wnt pathway in IBD patients results in Paneth cell dysfunction and finally destroys the intestinal mucosal barrier. Wnt pathway-related genes showed differential expression patterns in IBD patients compared with healthy controls [99]. In CD, the expression of TCF1 and TCF4 was reduced, and the defensin secretion was subsequently inhibited, which hindered the repair of the intestinal barrier. TCF1 and TCF4 regulated the production of defensins in Paneth cells [100]. The accumulation of β-catenin was detected in both the cytoplasm and nucleus of colon tissues from UC patients, but not in CD samples [101]. However, many studies found that β-catenin protein and mRNA expression and nuclear translocation of β-catenin were decreased after DSS treatment in mouse models [102,103]. The DSS-induced colitis model is one of the most widely used preclinical models for studying IBD pathogenesis. In this model, administration of DSS in drinking water induces epithelial barrier disruption, innate immune activation, and subsequent development of colitis characterized by symptoms resembling human IBD [104]. Nevertheless, DSS-induced colitis differs from human IBD in several fundamental aspects. Notably, its aetiology is characterized by direct chemical epithelial injury rather than the complex, multifactorial aetiology of human IBD, which might explain the differences in β-catenin expression profiles between human IBD and DSS-induced colitis models. Interestingly, the role of Wnt5a in inflammation is controversial [105,106]. Akira Sato and colleagues demonstrated that conditional knockout of Wnt5a in mice reduced pro-inflammatory cytokine production and alleviated DSS-induced inflammation [107]. However, another research in 2022 found that Wnt5a peptide suppressed the colitis induced by DSS in mice and downregulated the mRNA level of TNFα and IL-8 [108]. Interestingly, UC patients with subsequent relapses showed lower expression of Wnt5a mRNA than patients who remained in the remission stage [108].

CircRNAs have been validated to regulate intestinal homoeostasis through the Wnt pathway. CircRNA_Maml2 expression was significantly decreased in the intestinal tissues of severe burn mice and patients compared with normal controls [109]. Ectopic expression of circRNA_Maml2 promoted the proliferation and migration of murine colon carcinoma CT26 cells in vitro and alleviated intestinal mucosal inflammation after severe burns in mice. Interestingly, circRNA_maml2 level was positively correlated with FZD7, but negatively correlated with miR-93-3p. CircRNA_Maml2 upregulated FZD7 by sponging miR-93-3p and subsequently activated the Wnt/β-catenin pathway to promote the repair of the damaged intestinal mucosa in mice. However, the function and mechanism of circRNA_Maml2 need further exploration in humans. Wan D et al.also found that mmu_circ_0001845 was highly expressed in CAC tissues compared to the normal and colitis tissues [64]. Mmu_circ_0001845 expression paralleled with β-catenin and forced expression of mmu_circ_0001845 increased the nuclear accumulation of β-catenin and promoted the TOP/FOP luciferase activity. Interestingly, the human homologue of mouse mmu_circ_0001845, including hsa_circ_0124022, hsa_circ_0124028 and hsa_circ_0124029, exhibited higher expression levels in CAC patients than in UC and healthy ones. The expression of hsa_circ_0124022, hsa_circ_0124028 and hsa_circ_0124029 in CAC patients was associated with the duration from the UC onset to carcinoma, TNM stage, and clinical prognosis, which suggested that these circRNAs might be novel biomarkers for distinguishing CAC patients from UC and healthy ones, prognostic markers, and even promising therapeutic targets for CAC. Furthermore, these three circRNAs also promoted the colitis-to-carcinoma transformation through the Wnt/β-catenin pathway.

ISCs localize at the base of intestinal crypts and have the ability to self-renew and differentiate into multiple lineages [110]. There are two types of ISCs: active crypt base columnar cells marked by Lgr5 and quiescent +4 cells marked by Bmi1. The regulation of ISC homoeostasis plays a vital role in intestinal regeneration [111]. It has been proven that circRNAs play a critical role in regulating self-renewal maintenance of ISCs [112]. High expression of circPan3 was detected in both mouse and human Lgr5+ ISCs, and circPan3 knockdown impeded the self-renewal of Lgr5+ ISCs and epithelial regeneration depending on immune cells [113]. CircPan3 binds to and stabilizes IL-13ra1 mRNA, which encodes and expresses IL-13Rα1 (the IL-13 receptor subunit) in Lgr5+ ISCs. Upon receiving IL-13 secreted by group 2 innate lymphoid cells in the niche, STAT6 is activated, subsequently initiating Foxp1 expression. Finally, Foxp1 binds to and facilitates the nuclear translocation of β-catenin, activating Wnt/β-catenin signalling in Lgr5+ ISCs. Yu et al. reported that the intestinal barrier was disrupted and the number of Lgr5+ ISCs was decreased in the DSS-induced IBD mouse model [114]. Intravenous injections of human ASCs-derived exosomes into the above model reduced proinflammatory cytokines, promoted the regeneration of Lgr5+ ISCs, and protected the integrity of the gut barrier. Furthermore, ASCs-derived exosomes promoted the growth and regeneration of colon organoids and alleviated TNF-α-induced inflammation in colon organoids. Considering the function and mechanism of circPan3 in ISCs, it can be postulated that circPan3 may play a role in the pathophysiology of IBD, but further research is needed.

MSCs repair tissues and suppress inflammation via regulating immune homoeostasis and regeneration function [115,116]. MSCs increased the infiltration of T-reg cells in the intestine, inhibited dendritic cell maturation, and promoted the secretion of anti-inflammatory cytokines. MSCs might be isolated from many sources, including adipose, bone marrow, umbilical cord and so on, which were named respectively as follows: adipose-derived stem cells (ASCs), bone marrow-derived MSCs (BM-MSCs), and umbilical cord-derived MSCs (UC-MSCs). Many clinical trials of allogeneic or autologous ASCs, BM-MSCs, UC-MSCs, or haematopoietic stem cells (HSCs) therapy for CD-associated perianal fistula, refractory CD, or UC have been conducted and shown clinical response or even complete healing [117–121]. The delivery methods of MSCs included intralesional injection and intravenous administration. There were no severe adverse events in the follow-up study. The MSCs therapy might be a safe and effective option for refractory UC, CD, and CD-related fistula [122]. MSC-derived extracellular vesicles (MSC-EVs), such as exosomes (MSC-Exos), not only possess immunomodulatory characteristics similar to MSCs but also have the advantages of high stability and low immunogenicity [123,124]. In addition, circRNAs are enriched and stably expressed within exosomes, and can mediate intercellular signalling regulation via exosomal transport. Moreover, MSC-EVs have been validated to be a promising delivery system for both drugs and circRNA. The phase I clinical trial of MSC-Exos for the treatment of refractory perianal fistula in CD showed that three of the five patients who received MSC-Exos injection achieved complete healing of fistula, and all patients had no adverse events [125]. The efficacy of MSC-EVs for DSS-induced colitis in mice was also confirmed in mice [126–128]. A larger sample, multi-centre randomized controlled study and long-term follow-up are needed to further validate the efficacy and safety of MSC-EVs therapy in IBD.

Wnt signalling has been identified as a key factor in the fate decision and differentiation of MSCs [129]. Many studies have shown that circRNAs are closely associated with the regulation and function of MSCs. Interestingly, hsa_circ_0001320 (circFOXP1) expression was increased in MSCs and decreased during differentiation [130]. Knockdown of circFOXP1 reduced the expression of MSCs markers including CD164, PDPN, CD146 and GLI1, and decreased MSC differentiation capacity. Mechanistically, circFOXP1 maintained MSC differentiation capacity depending on miR-17-3p/miR-127-5p through EGFR and non-canonical Wnt signalling, which were associated with MSC capacity maintenance. Moreover, circFOXP1 and non-canonical Wnt5a were highly expressed in MSCs, while canonical Wnt3a was downregulated. Reprogramming MSCs towards pluripotency decreased circFOXP1 and non-canonical Wnt5a, but upregulated canonical Wnt3a. Overall, circFOXP1 was necessary for MSC identity and differentiation, while whether circFOXP1 is involved in the pathological process of IBD is worth exploring and may broaden our understanding of MSCs.

In addition, Wnt pathway mutations, such as APC mutations, GSK3 deletions, and β-catenin mutations, are often found in various cancers, including colorectal cancer (CRC) [,^86^,^131^]. Most sporadic CRC cases result from APC allele deletion, especially in the family of adenomatous polyposis. Loss of APC alleles inhibits the formation of the destruction complex, which leads to excessive accumulation of β-catenin and finally results in CRC. However, the pathophysiology of CRC and CAC shows different patterns. In contrast to sporadic CRC, β-catenin mutations are rarer, and APC mutations are less frequent and occur later in CAC, whereas p53 and K-ras mutations occur earlier. Reactive oxygen species (ROS) production and intestinal microbiome also play important roles in the pathogenesis of CAC [132].

Overall, the Wnt pathway might be a potential target for the treatment of IBD and IBD-related fibrosis. However, targeting the Wnt pathway may represent a double-edged sword, requiring a precise balancing, given its pivotal roles in stemness maintenance and tissue homoeostasis [133]. Although inhibitors or agonists of the Wnt pathway have been applied in experimental or cellular models, they have not yet been approved for clinical application [[89]].

## Other pathways

In addition to the JAK/STAT and Wnt pathways, circRNAs are also associated with other signalling pathways, including the PI3K/AKT, NF-κB, and Hippo pathways. The PI3K/AKT pathway is important in cell proliferation, apoptosis, metabolism, and inflammation regulation [134]. Generally, this pathway is initiated by various factors, such as cytokines, growth factors, and insulin. Subsequently, PI3K is activated and phosphorylates its substrates, which in turn phosphorylate and activate serine kinase AKT (also known as protein kinase B). Activated AKT then phosphorylates and inhibits the downstream substrates, including GSK3, forkhead box O (FOXO) transcription factors, and tuberous sclerosis complex 1/2 (TSC1/2). For instance, AKT activation inhibits GSK3, which is responsible for the degradation of β-catenin, thereby activating the Wnt/β-catenin pathway. Activated AKT activates the mammalian target of rapamycin complex 1 (mTORC1) by inhibiting TSC1/2, to regulate cell survival, autophagy, and protein synthesis. The hyperactivation of epithelial mTOR can result in the necroptosis of IECs and the dysfunction of Paneth cells, which in turn predisposes to severe colitis. Phosphatase and tensin homolog (PTEN), a well-known negative regulator of PI3K, inhibits AKT activation. LY294002, wortmannin, and rapamycin are well-characterized inhibitors of the PI3K/AKT pathway. The PI3K/AKT pathway may regulate the ROS homoeostasis, IECs apoptosis, and immune cells activation and polarization, which are closely related to the pathogenesis of IBD [135–138]. Moreover, NF-κB is a critical transcriptional regulator in modulating inflammation and immune responses. AKT can activate the NF-κB pathway by phosphorylating the IκB kinase to promote the production of pro-inflammatory cytokines, including TNF-α, IL-1β, and IL-12, which are also crucial in IBD [139]. Although inhibitors of the PI3K/AKT pathway, including alpelisib, inavolisib, and capivasertib, have been approved for advanced breast cancer [140], therapeutic targeting of this pathway remains unexplored in the context of IBD, with no approved agents currently available for IBD treatment.

Many circRNAs have been demonstrated to exert their functions in various diseases through the pathways mentioned above, including cancer and IBD [141,142]. In CD patients’ PBMCs, hsa_circRNA_103124 expression was upregulated and had a positive correlation with the white blood cell (WBC) count, faecal calprotectin level, TNF-α, and Crohn’s disease activity index (CDAI) [51]. Hsa_circRNA_103124 promoted intestinal epithelial cell proliferation and inhibited autophagy through sponging hsa-miR-650 and subsequently targeting the AKT2/CDK2 and AKT2/TSC1 pathways. On the other hand, hsa_circRNA_103124 promoted macrophage M1 polarization by activating AKT2 and the toll-like receptor 4 (TLR4)/NF-κB pathways, which increased the permeability of the intestinal epithelium [50]. TLR4 is a transmembrane protein that is crucial in pathogen recognition and innate immune activation. Upon activation by lipopolysaccharide (LPS), TLR4 recruits myeloid differentiation factor 88 (MYD88) and subsequently activates NF-κB. The expression of TLR4 is high in IBD, leading to the over-activation of downstream signalling and overgeneration of inflammatory cytokines [143–145]. Moreover, hsa_circRNA_103124 was also found to be highly expressed in colonic tissues and serum exosomes of UC patients [52]. Knockdown of hsa_circRNA_103124 suppressed TNF-α-induced cell apoptosis, inflammation, and oxidative stress through the miR-1236-3p/MYD88 axis. Thereby, hsa_circRNA_103124 might be a potential biomarker for the diagnosis and treatment of IBD.

The Hippo pathway is evolutionally conserved and has been implicated in IBD through regulating the immune response, inflammation, angiogenesis, intestinal epithelial regeneration, and gut microbiota [146,147]. When the Hippo pathway is off, the downstream transcriptional coregulators Yes-associated protein (YAP) and transcriptional coactivator with PDZ-binding motif (TAZ) translocate to the nucleus, where they initiate the transcription of target genes to modulate cell proliferation, stemness, and proliferation. Once the Hippo pathway is activated, YAP/TAZ is phosphorylated and inhibited by the upstream kinases, which in turn suppresses the transcription of downstream genes.

The upregulation of hsa_circRNA_104507 (circHIPK2) was detected in colon tissues of the patients with IBD and CRC, and DSS-treated colon tissues in mice model [56]. Knockdown of circHIPK2 aggravated DSS-induced colitis but suppressed the proliferation of CRC cells. CircHIPK2 enhanced the translational efficiency of TAZ and increased the transcription of target genes, namely cellular communication network factor 1 (CCN1) and CCN2, leading to intestinal epithelial growth in colitis and CRC. Considering the bidirectional effects of circHIPK2 on colitis and CRC, the application of circHIPK2 in IBD treatment may result in adverse events, and further exploration is required. In the septic rats model, circ_0001105 expression was decreased, while the levels of TNF-α, IL-6, and IL-1β were elevated [148]. The forced expression of circ_0001105 reduced intestinal mucosal permeability, oxidative stress, inflammatory cytokines and YAP1 expression. In addition, circRNAs also participate in CRC progression via the Hippo pathway, including circPPP1R12A, circ0106714, and hsa_circ_0128846 [149–151].

**Figure 2.**
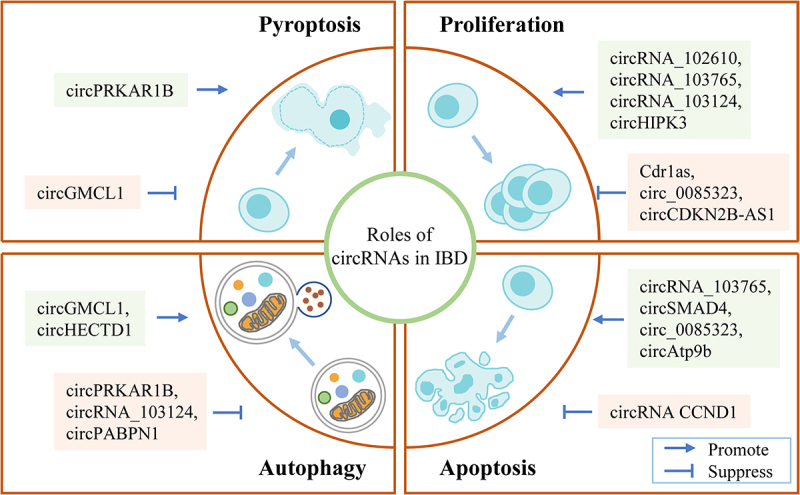
The roles of circRNAs in IBD. CircRNAs regulate cell proliferation, apoptosis, autophagy, and pyroptosis in IBD.

In addition, emerging evidence has highlighted the critical roles of autophagy and inflammasome-mediated pyroptosis in IBD pathogenesis, with circRNAs identified as key regulators of these processes (Figure 2) [40,48,51,58,63,152]. CircGMCL1 was significantly downregulated in colon tissues of CD patients and negatively correlated with CDAI [40]. CircGMCL1 protected against colitis by alleviating NLRP3 inflammasome-induced pyroptosis through promoting autophagy via the miR-124-3p/Annexin 7 axis in epithelial cells. Moreover, PLGA MSs carrying oe-circGMCL1 demonstrated therapeutic efficacy in IL-10 knock-out mice. Conversely, circPRKAR1B, upregulated in CD colon tissues, exacerbated colitis by suppressing autophagy and aggravating NLRP3 inflammasome-induced pyroptosis in both epithelial cells and IL-10-deficient mice [48]. Collectively, these findings show that circRNAs serve as crucial modulators of autophagy and NLRP3 inflammasome-mediated pyroptosis in IBD. The successful delivery of oe-circGMCL1 via PLGA MSs further highlights the therapeutic potential of circRNA-based strategies for IBD.

## Discussion

This review systematically integrates the crosstalk between circRNAs and classic signalling cascades, including JAK/STAT, Wnt, PI3K/AKT, NF-κB, and Hippo, in IBD. These pathways collectively govern intestinal inflammation, epithelial barrier integrity, mucosal repair, stem-cell regulation, fibrosis, and CAC. Accumulating evidence indicates that circRNAs, rather than being non-functional splicing byproducts, function as dynamic regulators in IBD biology, exerting either protective or pathogenic effects depending on the cellular and temporal contexts of the disease. We have not only elucidated the functional diversity of circRNAs but also highlighted their potential as biomarkers and therapeutic targets.

CircRNAs are involved in several key aspects of IBD pathology, including intestinal inflammation, epithelial barrier dysfunction, mucosal repair, stem-cell regulation, fibrosis, and CAC. CircSMAD4 promoted intestinal inflammation and IEC apoptosis, and impaired the intestinal barrier through the miR-135a-5p/JAK2/STAT3 axis [47]. Other circRNAs such as circPRKAR1B, circGMCL1, circRNA_103124, circRNA_103516, circHIPK2, circRNA CCND1, circHECTD1, and circ_0085323 regulate intestinal inflammation, whereas circGMCL1, circSOD2, circHIPK3, hsa_circ_0001021, hsa_circ_0007919, circCDKN2B-AS1, and circPABPN1 are involved in maintaining epithelial barrier integrity. CircPan3 promoted the self-renewal of Lgr5+ ISCs and epithelial regeneration through Wnt/β-catenin signalling [113]. In contrast, circBtnl1, highly expressed in ISCs, suppressed the self-renewal of ISCs through interacting with Ddx3y to destabilize Atf4 mRNA and inhibit Sox9 transcription [112]. CircFOXP1 maintained MSC differentiation capacity via sponging miR-17-3p/miR-127-5p, thereby regulating EGFR and non-canonical Wnt pathways [130]. For fibrosis, circRNA_102610, upregulated in PBMCs from CD patients, promoted proliferation of IECs and TGF-β1-induced epithelial–mesenchymal transition (EMT), a process contributing to CD-related fibrosis, via sponging hsa-miR-130a-3p [45]. Mmu_circ_0001845 promoted colitis-to-carcinoma transformation through the Wnt/β-catenin pathway [64]. Moreover, circRNAs serve as central nodes that coordinate multiple pathways simultaneously. For example, circRNA_103124 promoted macrophage M1 polarization and intestinal inflammation through both the AKT2 and TLR4/NF-κB pathways, linking inflammatory signalling to immune cell modulation [50]. Mmu_circ_0001109 aggravated colitis through JAK/STAT and NF-κB pathways, illustrating crosstalk between two major inflammatory cascades [64].

The most classic mechanism of circRNAs is functioning as ceRNAs to sponge miRNAs and upregulate the target genes. Many studies have preliminarily explored the characteristics and functions of synthetic circRNAs containing multiple miRNA binding sites. For example, artificial circRNAs incorporating miR-21 or both miR-21 and miR-93 binding sites have shown suppressive effects on cancer cell proliferation and migration, with greater stability than linear counterparts [153,154]. Given that certain miRNAs are dysregulated in IBD and regulate intestinal homoeostasis, we propose exploring artificial circRNAs containing target miRNA binding sites as a potential therapeutic strategy for IBD [155]. CircRNAs function through multiple mechanisms beyond miRNA sponging, including mRNA stabilization, translational regulation, and RBPs interactions. For example, circPan3 promoted the self-renewal of Lgr5+ ISCs and epithelial regeneration through binding to and stabilizing IL-13ra1 mRNA [113]. CircHIPK2 promoted the translational efficiency of TAZ and increased the transcription of target genes to increase intestinal epithelial growth in colitis and alleviate colitis [56]. CircPABPN1, upregulated in colon tissues of UC patients, regulated autophagy through blocking the binding of the RBP HuR to Atg16l1 mRNA and suppressing ATG16L1 translation in the intestinal epithelium [63]. Similarly, circPRKAR1B interacted with the RBP spectrin beta, non-erythrocytic 1 (SPTBN1), leading to inhibition of autophagy and aggravation of pyroptosis in epithelial cells [48].

Their structural stability and tissue-specific expression pattern position circRNAs as promising diagnostic biomarkers and therapeutic targets for IBD. CircRNA_092520, circRNA_102610, circRNA_004662, and circRNA_103124 were significantly upregulated in PBMCs of CD patients, with AUC values of 0.66, 0.78, 0.85, and 0.74, respectively. Notably, circRNA_004662 may be a preferable diagnostic biomarker for CD and for distinguishing CD from UC [43]. CircRNA_103765, which was upregulated in CD patients’ PBMCs, promoted IECs proliferation and apoptosis via the miR-30/DLL4 pathway, serving as a potential diagnostic marker and therapeutic target [46]. In addition, circRNA_102685, hsa_circ_0001666, hsa_circ_0062142, circRNA_103516, hsa_circ_0007919, hsa_circ_0085323, and circAtp9b have also been identified as valuable diagnostic markers for IBD [42,49,53,60,62,65]. Moreover, circSMAD4, circPRKAR1B, circGMCL1, Cdr1as, circHIPK2, circHIPK3, circRNA CCND1, circHECTD1, hsa_circ_0001021, and circCDKN2B-AS1 may represent promising therapeutic targets for IBD [40,41,47,48,55–59,61].

The high stability and long half-life of circRNAs make them attractive candidates for vaccine and drug development. Engineered circRNAs encoding relevant antigens have demonstrated prophylactic and therapeutic effects in animal models of melanoma and COVID-19, with advantages in stability and durability over mRNA vaccines [156,157]. However, the circularization efficiency, nucleotide modifications, purification, and delivery of circRNA vaccines need to be further investigated. The efficacy and safety of circRNA vaccines await further exploration in clinical trials.

The emerging recognition of circular RNAs (circRNAs) as pivotal regulators of intestinal homoeostasis and inflammatory responses has positioned them as promising therapeutic targets for IBD. Nevertheless, the journey of circRNAs from bench to bedside remains impeded by several obstacles (Table 2). First, current circRNA research in the context of IBD remains predominantly restricted to single cell lines and preclinical animal models, lacking comprehensive validation across diverse cellular contexts and systemic physiological effects. The transition from monocellular systems to multicellular organoids and organ-on-chip platforms may offer a promising intermediate step towards clinical fidelity, facilitating the precise identification of disease-relevant circRNA candidates. Second, although PLGA microspheres enable colon-targeted delivery, optimizing lipid nanoparticle formulations for circRNA encapsulation, minimizing off-target effects and enhancing biosafety remain critical challenges. Third, although the structural stability of circRNAs may confer prolonged pharmacodynamic duration, whether this attribute translates into more persistent or irreversible adverse effects remains poorly characterized. A concerted effort to resolve these technical and translational obstacles will be pivotal in accelerating the clinical translation of circRNA-based strategies for IBD management.

**Table 2.** CircRNAs in IBD pathways: from current evidence to clinical application.

Pathway	Representative circRNA	Evidence source	Main limitation	Future direction	Reference
JAK/STAT	circSMAD4	CircSMAD4, highly expressed in colon tissues of CD patients, impaired intestinal barrier integrity and promoted colitis progression via the miR-135a-5p/JAK2 axis in epithelial cells. PLGA MSs carrying si-circSMAD4 treatment alleviated intestinal inflammation in IL-10 knockout mice.	1. Best circRNA target not confirmed 2. Single Chinese CD cohort 3. Only JAK2/STAT3 examined 4. Delivery method not yet optimized for humans 5. Only two cell lines tested	1. Screen more circRNAs in larger cohorts 2.Validate in multi-ethnic, multi-centre cohorts 3. Explore additional mechanisms 4. Explore appropriate dosages and local delivery approaches 5. Validate in patient-derived intestinal organoids and and other intestinal cell types	[47]
Wnt	circPan3	CircPan3 promoted the self-renewal of mouse Lgr5+ ISCs and epithelial regeneration through Wnt/β-catenin signalling, which was further validated in circPan3-deficient mice.	1. Function and mechanism validated only for mouse circPan3, human circPAN3 lacks data (expression only) 2. Role and mechanism of circPAN3 in IBD pathogenesis unexplored	Validate the function and mechanism of human circPAN3 in IBD	[113]
PI3K/AKT	circAtp9b	CircAtp9b, upregulated in the plasma samples of UC patients, promoted LPS-induced apoptosis via PTEN in human colonic epithelial cells.	1. Small sample size; single-centre; no disease activity correlation; only plasma, not colonic tissue 2. No animal model validation	1. Validate in larger, multi-centre cohorts; measure expression in colonic biopsies; correlate with clinical parameters 2. Validate in circAtp9b-knockdown or knockout mouse model	[65]
NF-κB	circRNA_103124	CircRNA_103124 promoted macrophage M1 polarization and intestinal inflammation through both the AKT2 and TLR4/NF-κB pathways in THP1 cells.	Lack of in vivo animal model validation	Construct a circRNA-overexpressing or knockdown mouse model	[50]
Hippo	circHIPK2	CircHIPK2, upregulated in colon tissues of both IBD and CRC, alleviated colitis but promoted colitis-associated tumorigenesis through enhancing TAZ translation in vitro, in the colitis mice model, and the colitis-associated tumour mice model.	1. Small human cohort 2. Role of circHIPK2 in other cell types unexplored	1. Validate in larger, multi-centre cohorts 2. Explore the function of circHIPK2 in other cell types, such as immune cells	[56]

In summary, circRNAs play important roles in IBD pathogenesis and may serve as promising diagnostic markers, prognostic predictors, and therapeutic targets of IBD. Further research on circRNAs is needed to pioneer new directions for IBD treatment.

## Data Availability

Data sharing is not applicable to this article as no new data were created or analysed in this study. All information synthesized in this review was obtained from published articles, which are cited in the reference list. Further inquiries can be directed to the corresponding author (chenghistory@tmmu.edu.cn).
